# Chronic Myelogenous Leukemia (CML) in the elderly

**DOI:** 10.4084/MJHID.2014.037

**Published:** 2014-06-01

**Authors:** Lodovico Balducci, Dawn Dolan

**Affiliations:** H. Lee Moffitt Cancer Center and Research Institute. 12902 Magnolia Dr, Tampa, Florida 33612.

Approximately 50 % of CML patients are 66 and older.^1^ In earlier studies age appeared a poor prognostic factor for survival and was included as such in all staging models.^2^ It is not clear whether this age effect was due to poorer disease biology, or to other age related factors, including competitive causes of death, increased risk of treatment-related complications, or contraindications to curative bone marrow transplant. The introduction of tyrosine kinase inhibitors (TKI) that are better tolerated than previous treatments and that prolong the survival of the majority of responding patients indefinitely, allow a fresh look to the interactions of advanced age and CML. The issue is timely as one expect that the median age of CML and the prevalence of the disease in older individuals will progressively increase with the aging of the population.^3^

After reviewing the definition of age in physiologic terms, this editorial explores the biology of CML, the effectiveness and tolerance of TKI in older people and the age adjusted survival of CML patients since the introduction of TKI.

## Physiologic and Chronologic Age

Aging is associated with a progressive reduction in life-expectancy and functional reserve, and with increased polymorbidity. The ability of independent living of the older person may be impaired by loss of memory and judgment and by inadequate sight and hearing. The ability of independent living and stress tolerance may be determined by the availability of adequate social support.^4^ Though these changes are universal they occur at different rate in different individuals, so that chronologic age reflects chronologic age poorly. Individualized treatment of cancer in the older aged person implies an estimate of life expectancy and of the risk of treatment related complications. A number of laboratory tests have been proposed to determine physiologic age including the inflammatory index^4^, the length of leukocyte telomers,^5^ and the expression of the gatekeeper gene PINK4a,^6^ in normal tissues. None of these tests has been validated in patients with neoplastic diseases, however. It was repeatedly demonstrated that a Comprehensive Geriatric Assessment (CGA) may estimate both mortality risk and tolerance of antineoplastic treatment.^3^ The CGA involves evaluation of function, comorbidity, polypharmacy, cognition, depression, social support, and economic issues. It is recommended that some form of CGA be performed in all individuals aged 70 and older.^7^ The assessment of physiologic age may provide the answer to important questions related to the management of CML in the elderly and in particular whether the cancer independent life-expectancy is long enough to justify the initiation of any treatment, whether the patients may tolerate treatment and whether the patients pharmacopeia needs adjustment to prevent dangerous drug interactions. In countries without a national health care system the CGA may also reveal whether the patient may afford treatment.

## Biology of CML and Aging

Though a number of early studies summarized in reference 2 demonstrated that the advanced age was an independent prognostic factor for poor survival, none of them demonstrated a more advanced disease stage at presentation, or a higher prevalence of multiple cytogenetic abnormalities in older individuals. Likewise, more recent studies failed to demonstrate an association of age with BCR/abl mutations purporting TKI resistance.^8^ While it is still possible that age-associated immune dysfunctions or age-related changes in hematopoietic stroma may lead to a more aggressive disease, at present there is no conclusive evidence that the biology of CML changes with age. As older patients appear to benefit from TKI to the same extent as the young^1^,^9^–^10^ one may conclude that age-related biological differences do not seem to impact the prognosis of CML in the elderly.

## Age and Survival of CML Patients in the Imatinib Era

Two large population studies^1^,^9^ have examined the changes in CML survival among patients of all ages, since the advent of imatinib. The Surveillance, Epidemiology, and End Results (SEER) study showed that the survival of patients in all age groups had improved to the same extent^1^. A Swedish study encompassing all patients treated in the country since 1958 (when the national tumor registry was established) until 2008 showed that the survival of all patients had improved except for those aged 79+. The causes of this age-related discrepancy could not be analyzed based on the registry data. It is not farfetched to assume that competitive causes of death and inadequate treatment might have played a major role. An Italian study involving 31 medical centers^10^ showed that patients aged 75+ seemed to benefit from imatinib treatment to the same extent as younger people. It is not clear to what degree these patients have been selected however.

## Age and Pharmacology of TKI

Most of the information available concerns imatinib. In clinical studies the risk^8^ of imatinib related side effects and in particular of fluid retention increased after age 65. Also the combination of imatinib with p450 inhibitors should be used with caution and the combinations of these agents with desatinib and nilotinib should be avoided. P450 inhibitors decrease the metabolism of TKI and may enhance their complications. The Italian study^10^ demonstrated that the achievement of complete cytogenetic response was similar in individuals 75 and older and younger individuals. However a major molecular response was rarer. Also the AUC of imatinib was increased and the elimination decreased in the elderly. These individuals required more frequent dose reductions or treatment interruptions than the young ones.

## Conclusions

Current evidence suggests that the biology of CML is not affected by patient’s age and that older individuals may benefit from TKI treatment to the same extent as the younger ones. Older individuals may require more frequent dose reductions, as well as pharmacologic adjustments, to avoid drug interactions. The assessment of physiologic age through a CGA may reveal the patients more likely to benefit from treatment. The cost of these agents may represent a treatment barrier for older individuals living in countries without universal health care.
